# *CTLA4* DNA methylation is associated with CTLA-4 expression and predicts response to immunotherapy in head and neck squamous cell carcinoma

**DOI:** 10.1186/s13148-023-01525-6

**Published:** 2023-07-06

**Authors:** Friederike Hoffmann, Alina Franzen, Luka de Vos, Lennert Wuest, Zsófi Kulcsár, Simon Fietz, Alexander Philippe Maas, Sarah Hollick, Marie Yatou Diop, Jennis Gabrielpillai, Timo Vogt, Pia Kuster, Romina Zarbl, Joern Dietrich, Glen Kristiansen, Peter Brossart, Jennifer Landsberg, Sebastian Strieth, Dimo Dietrich

**Affiliations:** 1 Department of Dermatology and Allergy, University Medical Center Bonn, Bonn, Germany; 2 Department of Otorhinolaryngology, University Medical Center Bonn, Venusberg–Campus 1, 53127 Bonn, Germany; 3 Institute of Pathology, University Medical Center Bonn, Bonn, Germany; 4 Department of Oncology, Hematology and Rheumatology, University Medical Center Bonn, Bonn, Germany

**Keywords:** *CTLA4*, CTLA-4, Head and Neck Squamous Cell Carcinoma (HNSCC), DNA methylation, Biomarker, Immunotherapy, Anti-PD-1

## Abstract

**Background:**

The majority of patients with recurrent or metastasized head and neck squamous cell carcinoma (HNSCC) do not benefit from immune checkpoint blockade (ICB) while several patients experience severe and persistent immune-mediated side effects. Therefore, predictive biomarkers are urgently needed to allow for a personalized treatment. In this study, we investigated DNA methylation of the immune checkpoint gene *CTLA4* with regard to its predictive value.

**Methods:**

We analyzed *CTLA4* promoter methylation in tumors of HNSCC patients (*N* = 29) treated with ICB at the University Medical Center Bonn with regard to response to ICB and progression-free survival. We further analyzed a second cohort (*N* = 138) of patients that did not receive ICB with regard to *CTLA4* promoter methylation, CTLA-4 protein expression, and immune cell infiltrates. Finally, we tested inducibility of CTLA-4 protein expression in HNSCC cells using the DNA methyltransferase inhibitor decitabine.

**Results:**

Lower *CTLA4* promoter methylation correlated with response to ICB and prolonged progression-free survival. We could show that not only tumor infiltrating immune cells, but also HNSCC cells harbor cytoplasmic and nuclear CTLA-4 expression. *CTLA4* promoter methylation inversely correlated with infiltrates of CD3^+^, CD4^+^, CD8^+^, and CD45^+^ immune cells. *CTLA4* methylation did not correlate with protein expression in tumors, however, decitabine treatment led to decreased *CTLA4* methylation and an induction of *CTLA4* mRNA and CTLA-4 protein expression in HNSCC cell lines.

**Conclusions:**

Our results indicate that *CTLA4* DNA hypomethylation is a predictive biomarker for response to ICB in HNSCC. Our study warrants further analyses of the predictive value of *CTLA4* DNA methylation in clinical trials of anti-PD-1 and/or anti-CTLA-4 immunotherapy in HNSCC.

## Background

Head and neck squamous cell carcinomas (HNSCCs) arise from the epithelium of the oral cavity, larynx, and pharynx and represent the most common cancers in the head and neck region [1]. It is an extremely heterogeneous disease, differing in risk factors, localization, etiology, oncogenic alterations, and prognosis [2, 3]. Although HNSCC has predominantly been correlated with exposure to tobacco carcinogens and alcohol consumption or both [4], a subset of HNSCC has been associated with human papillomavirus (HPV) infection [5]. Prognosis of HNSCC patients is poor, with a 5-year survival rate of ~ 65.0% [6]. Treatment options include surgical resection, radiation, radiation combined with cisplatin-based chemotherapy (chemoradiotherapy) or treatment with the monoclonal antibody cetuximab, alone or in combination with chemotherapy [7]. The inhibitors of the programmed cell death 1 (PD-1) receptor nivolumab and pembrolizumab have been approved for treatment of cisplatin-refractory HNSCC and additionally, pembrolizumab has been approved as first-line therapy in patients with locally advanced or metastatic disease [8, 9]. Combined immune checkpoint blockage (ICB) targeting the cytotoxic T lymphocyte–associated protein 4 (CTLA-4) and PD-1, has shown great efficacy in the treatment of metastasized malignant melanoma [10] and renal cell carcinoma [11]. CTLA-4 is expressed on the surface of activated T cells and functions as a negative regulator of T cell immune function [12]. Anti-CTLA-4 antibodies have not yet been approved for the treatment of HNSCC. However, there exist encouraging reports of HNSCC patients who were successfully treated with combined ICB. In the phase III trial CheckMate 651, treatment with nivolumab and the anti-CTLA-4 antibody ipilimumab led to prolonged overall survival and durable responses in patients with PD-1 ligand 1 (PD-L1) combined positive score (CPS) ≥ 20 and CPS ≥ 1 [13]. Furthermore, neoadjuvant treatment with nivolumab and ipilimumab of patients with locoregionally advanced HNSCC resulted in a major pathological response in 35% of the cases [14]. Treatment of HNSCC with anti-CTLA-4 antibodies as monotherapy or in combination with anti-PD-1 antibodies is currently investigated in various phase II/III trials (ClinicalTrials.gov Identifiers: NCT04080804, NCT04326257, NCT03624231, NCT03212469, NCT03799445; Table 1).

**Table 1:** Clinical trials investigating anti-CTLA-4 antibodies in head and neck squamous cell carcinoma (HNSCC)

Trial	Drug	Study phase	Tumor entity
NCT04080804	Neoadjuvant nivolumab (anti-PD-1) alone or in combination with relatlimab (anti-LAG3) or ipilimumab (anti-CTLA-4)	II	Resectable HNSCC
NCT03624231	Durvalumab (anti-PD-L1) + tremelimumab (anti-CTLA-4) + radiotherapy versus durvalumab (anti-PD-L1) + radiotherapy	II	Non-resectable locally advanced HPV negative HNSCC
NCT04326257	Nivolumab (anti-PD-1) + ipilimumab (anti-CTLA-4) or relatlimab (anti-LAG3)	II	Recurrent and/or metastastic HNSCC that have progressed on prior immunotherapy
NCT03212469	Durvalumab (anti-PD-L1) + tremelimumab (anti CTLA-4) in combination with stereotactic body radiotherapy	I/II	HNSCC
NCT03799445	Ipilimumab (anti-CTLA-4), nivolumab (anti-PD-1), and radiation therapy	II	HPV positive advanced oropharyngeal squamous cell carcinoma

Clinical trials investigating anti-CTLA-4 antibodies in monotherapy or in combination with other agents in head and neck squamous cell carcinoma (HNSCC). Listed are only phase II trials

Yet, despite the enormous achievements of ICB, most of the patients do not experience long-term remissions and some of them suffer from severe and persistent immune-mediated side effects [15]. Therefore, predictive biomarkers are desperately needed to identify patients that are most likely to benefit from immunotherapy. Currently, only PD-L1 expression has been shown clinical utility in HNSCC, as patients with a PD-L1 CPS ≥ 1 are more likely to respond to anti-PD-1 immunotherapy. Yet, the false positive predictive value remains high and also PD-L1 non-expressors have been shown to respond to PD-1/PD-L1 inhibitors at times [16].

Starzer et al. [17] could recently show that the DNA methylation profile is correlated with radiological response to anti-PD-1 ICB in HNSCC patients, suggesting that DNA methylation analysis may be helpful to predict response to ICB. Aberrant DNA methylation is an epigenetic hallmark of cancer [18] that can reliably be detected even in little quantities of formalin-fixed and paraffin-embedded (FFPE) tissues and therefore can function as a powerful biomarker. Numerous studies refer to aberrant methylation of immune checkpoint genes, i.e. *PD-1*, *PD-L1*, and *CTLA4* in various malignancies [19–23]. Moreover, we have shown that DNA methylation of C*TLA4* predicts response to anti-CTLA-4 and anti-PD-1 antibody therapy in melanoma and to anti-PD-1 ICB in renal cell carcinoma [21–23]. In order to test the relevance of *CTLA4* methylation in HNSCC, we analyzed methylation of these previously identified CpG sites in HNSCC cell lines and two cohorts of non-ICB treated and ICB treated HNSCC patients.

## Results

### ***CTLA4*** DNA methylation is inversely correlated with CD3^+^, CD4^+^, CD8^+^, and CD45^+^ immune infiltrates

We have recently reported a significantly lower *CTLA4* promoter methylation in isolated CD4^+^ and CD8^+^ T cells obtained from peripheral blood of healthy donors compared to HNSCC cell lines [24]. Also, our group described significantly lower CD4^+^, CD8^+^, and CD45^+^ infiltrates in *CTLA4* promoter hypermethylated clear cell renal cell carcinomas [23]. In order to investigate if the negative association between *CTLA4* promoter methylation and tumor immune infiltration is a tumor entity-independent phenomenon, we analyzed *CTLA4* promoter methylation and tumor-infiltrating immune cells in a cohort of *N* = 138 patients with HNSCC (UKB Non-ICB cohort) by means of quantitative methylation-specific PCR (qMSP). We observed significant but weak inverse correlations between *CTLA4* promoter methylation and presence of CD3^+^ (Spearman’s *ρ* =  − 0.19, *p* = 0.029), CD4^+^ (*ρ* =  − 0.23, *p* = 0.006), CD8^+^ (*ρ* =  − 0.17, *p* = 0.048), and CD45^+^ (*ρ* =  − 0.21, p = 0.014; all *N* = 138) immune cells within the HNSCC microenvironment.

### HNSCC cells show cytoplasmic and nuclear CTLA-4 expression

**Fig. 1:**
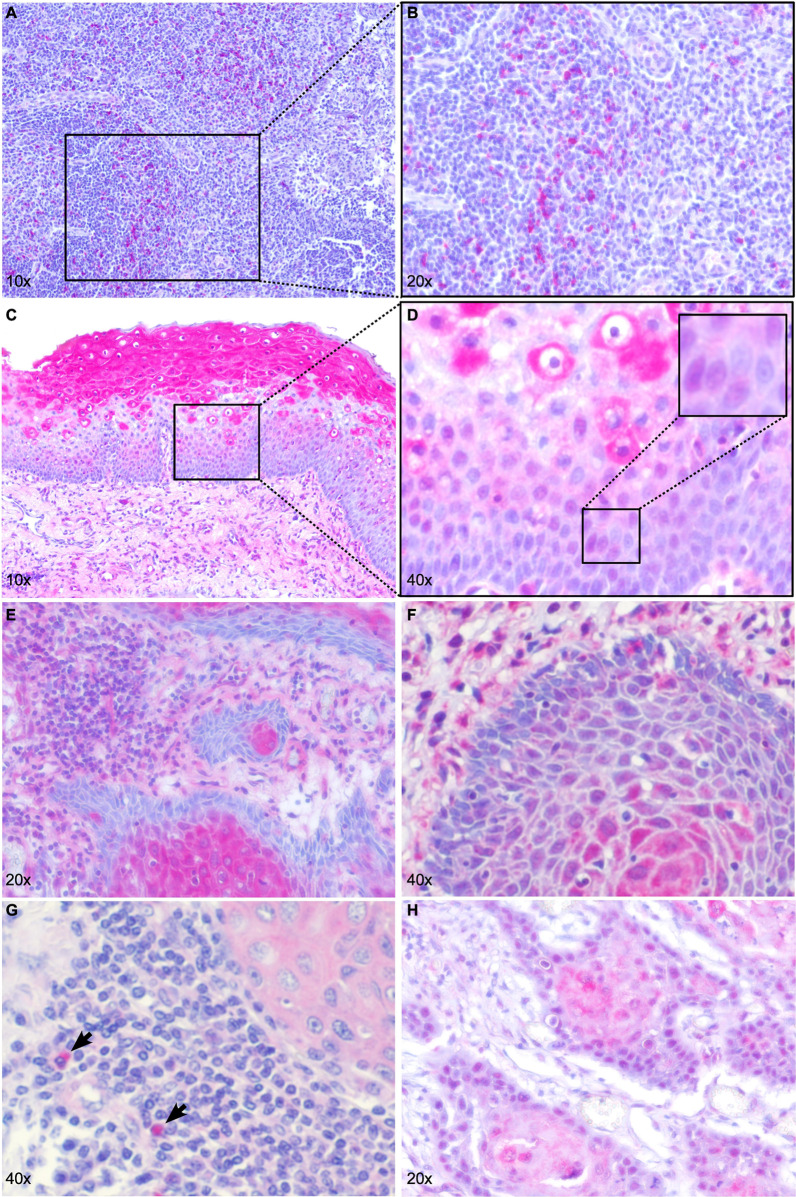
CTLA-4 protein expression in tonsillar and HNSCC tissue. Immunohistochemical staining of CTLA-4 in a tonsil (**a** in tenfold magnification, **b** in 20-fold magnification), tumor-associated squamous epithelium (**c** in tenfold magnification, **d** in 40-fold magnification) and exemplarily in four HNSCCs (**e–h** in 20-fold and 40-fold magnification, respectively). CTLA-4-expressing lymphocytes are present in tonsillar tissue (**a**, **b**). Keratinocytes of tumor-associated squamous epithelium show strong cytoplasmic and weak nuclear CTLA-4 expression (**c, d**). HNSCC with predominantly CTLA-4-positive lymphocytes. The keratinizing tumor nests show strongly CTLA-4-positive (cytoplasmic) centers and CTLA-4-negative peripheral tumor cells (**e**). HNSCC with similar CTLA-4 expression pattern compared to **e** and an additional nuclear expression by tumor cells (**f**). HNSCC with weakly CTLA-4-positive tumor and immune cells and sporadic strong positive lymphocytes (arrows, **g**). HNSCC with CTLA-4-positive tumor cells (nuclear and cytoplasmic) (**h**)

Our previous study revealed a large *CTLA4* promoter methylation variance among HNSCC cell lines, ranging from low to high levels [24]. We have shown CTLA-4 protein expression in melanoma cells and a negative correlation between *CTLA4* promoter methylation and *CTLA4* mRNA expression in melanomas [21, 22], therefore we hypothesized that CTLA-4 could also be expressed by HNSCC tumor cells. We analyzed HNSCC tumor cell-intrinsic CTLA-4 protein expression in the UKB Non-ICB cohort using a CE IVD-certified monoclonal antibody (clone BSB-88) as previously described [22]. We confirmed the specificity of the antibody in tonsillar tissue (Fig. 1a and b). Distinct cells stained strongly positive in the germinal centers close to the mantle zone. Strong positive CTLA-4 expression was as well observed in epithelial cells of tumor-associated epithelium (Fig. 1c and d), showing a gradient of CTLA-4-staining from basal to upper epidermis in accordance to the maturation process. Epithelial cells expressed CTLA-4 in the cytoplasm and to some extent also in the nucleus, the latter particularly in the stratum parabasale and stratum intermedium (Fig. 1d). HNSCCs showed a heterogeneous CTLA-4 expression pattern. Representative CTLA-4 expression patterns are shown in Fig. 1e–h. We found CTLA-4-positive and -negative lymphocytes as well as positive tumor cells (e.g. Figure 1e–h). Predominantly positive immune cells are shown in Fig. 1e and f. Figure 1g shows a tumor with mainly negative or weakly positive and sporadic strongly positive immune cells. Interestingly, in several tumors, CTLA-4 was also expressed by tumor cells (Fig. 1e–h). CTLA-4 expression followed a gradient from tumor periphery to the tumor center of keratinizing HNSCC (Fig. 1e, f), a gradient observed similarly to the adjacent epithelium. In most HNSCCs, CTLA-4 showed a cytoplasmic rather than membrane-bound expression (e.g., Figure 1g), which is in line with our previous findings from melanoma [22]. However, we observed several HNSCC cases in which CTLA-4 was expressed in the nucleus (Fig. 1f, h).

### CTLA-4 expression in HNSCC is inducible with the hypomethylating agent decitabine

**Fig. 2:**
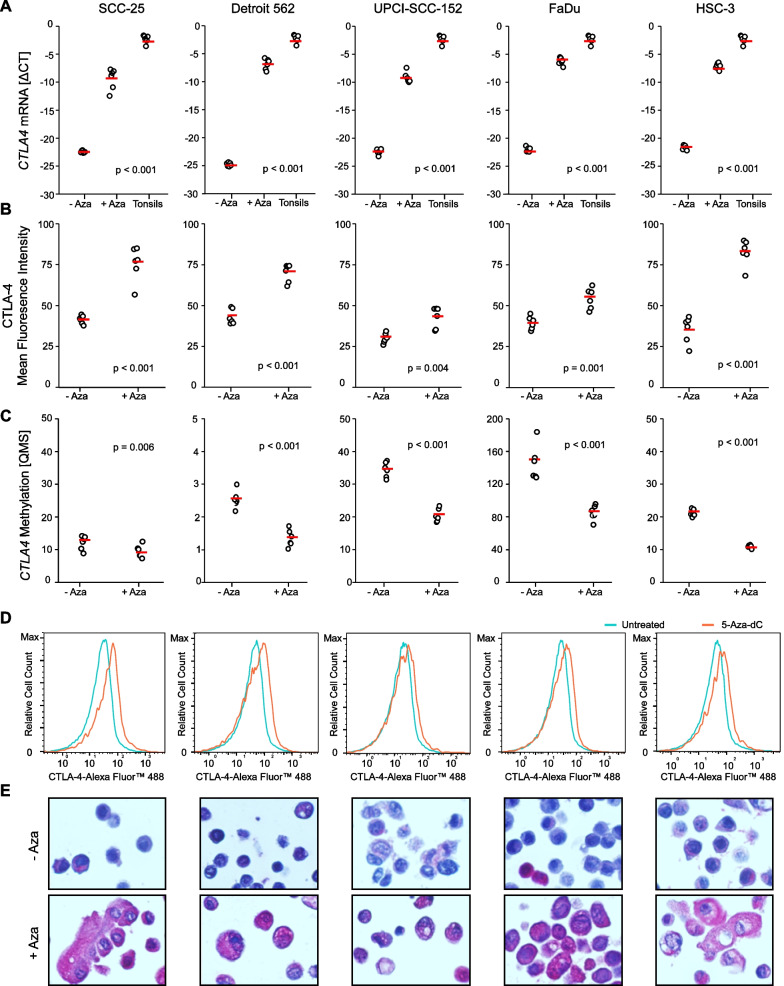
Pharmacological demethylation decreases *CTLA4* methylaton and induces CTLA-4 expression in head and neck squamous cell carcinoma (HNSCC) cells **a** *CTLA4* mRNA expression (ΔCT levels) in 5‐aza‐dC treated (+ Aza) compared to untreated (− Aza) HNSCC cells and six individual tonsils. **b** CTLA-4 expression (mean fluorescence intensity [MFI]) of 5-aza-dC treated (+ Aza) compared to untreated (− Aza) HNSCC cell lines. **c** *CTLA4* Methylation [QMS] of 5-aza-dC treated (+ Aza) compared to untreated (− Aza) HNSCC cell lines. **d, e** Normalized histograms (**d**) and IHC (**e**) illustrating induction of CTLA-4 protein expression in pharmacologically demethylated (+ Aza) compared to untreated (− Aza) HNSCC cell lines. Bars represent mean values. *P*-values refer to Kruskal–Wallis and *t*-tests, respectively

We have previously shown that in dependence on the sequence context of the analyzed CpG site, no or only weak correlations between *CTLA4* methylation and mRNA expression are present in HNSCC [24]. We correlated the tumor cell-intrinsic CTLA-4 protein expression quantified using the H scoring system with methylation levels in our UKB Non-ICB cohort. In concordance with our previous reports from HNSCC and melanoma [22, 24], we did not find significant correlations between *CTLA4* promoter methylation and CTLA-4 protein expression (*ρ* = -0.001, *p* = 0.99, *N* = 113). In the examined heterogeneous tumor samples, no correlations between *CTLA4* methylation and expression at mRNA or protein level were found. However, it is possible that since methylation analysis was not performed on a single cell level, a biologic connection is masked due to the heterogeneity of the sample. Therefore, we further analyzed the impact of DNA methylation on CTLA-4 expression in HNSCC in vitro. We treated five different human HNSCC cell lines with the hypomethylating agent decitabine and assessed *CTLA4* methylation via qMSP and CTLA-4 expression via quantitative reverse transcription PCR (qRT-PCR), flow cytometry, and immunohistochemistry (IHC) (Fig. 2). In the untreated cell lines, we detected only basal to absent CTLA-4 expression levels, even in hypomethylated cell lines like SCC-25 and Detroit 562. However, treatment with decitabine led to significantly decreased methylation levels of the analyzed CpG sites and an increase of *CTLA4* mRNA and CTLA-4 protein expression, albeit still on a significant lower level compared to tonsillar tissues, as shown in Fig. 2a–c. Treatment with decitabine led to morphological changes including cell enlargement with enlarged nuclei of the treated HNSCC cells.

### *CTLA4* promoter methylation predicts response to ICB and progression-free survival

Following, we investigated the prognostic and predictive value of *CTLA4* methylation in HNSCC. In Cox proportional hazards analysis a significant association between UICC stage and patients’ overall survival of *N* = 138 HNSCC patients that did not receive ICB (HR = 1.47 [95%CI 1.03–2.11], *p* = 0.036) was present, indicating the representative value of this small cohort. As already shown in the HNSCC cohort from TCGA [24], no association between *CTLA4* methylation and overall survival in this cohort could be observed (HR = 1.01 [95%CI 0.98–1.03], *p* = 0.67). Accordingly, *CTLA4* methylation was also not a significant prognostic parameter in multivariate Cox proportional hazards analysis of methylation and stage (*CTLA4* methylation: HR = 1.00 [95%CI 0.98–1.03], *p* = 0.73; UICC stage: HR = 1.44 [95%CI 1.01–2.05], *p* = 0.046).

**Fig. 3:**
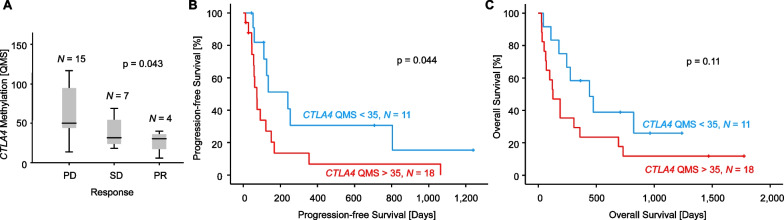
Association of *CTLA4* DNA methylation with ICB therapy response and patients’ survival in HNSCC. **a** Association of *CTLA4* DNA methylation with therapy response in a cohort of *N* = 29 ICB-treated HNSCC patients. Therapy response was grouped in progressive disease (PD), stable disease (SD), and partial response (PR). **b** Kaplan–Meier analysis of progression-free survival in HNSCC patients stratified according to *CTLA4* methylation in pre-ICB-treatment samples. **c** Kaplan–Meier analysis of overall survival in HNSCC patients stratified according to *CTLA4* methylation in pre-ICB-treatment samples. Patient samples were dichotomized based on an optimized cut-off

To investigate the predictive value of *CTLA4* DNA methylation in HNSCC, we examined *CTLA4* DNA methylation in tumor samples of *N* = 29 patients with advanced or metastatic HNSCC prior to ICB. Patients were treated with the anti-PD-1 inhibitors cemiplimab (*N* = 1), nivolumab (*N* = 27) or nivolumab combined with the anti-CTLA-4 inhibitor ipilimumab (*N* = 1). Therapy response data were reported based on RECIST criteria and were available for *N* = 26 patients. Fifteen of the patients suffered from progressive disease (PD), seven patients showed a stable disease (SD), and four patients had a partial response (PR) to ICB. *CTLA4* DNA hypermethylation significantly correlated with poor therapy response to ICB (*p* = 0.043; Fig. 3a).

Kaplan–Meier analyses of progression-free and overall survival of HNSCC patients stratified by *CTLA4* DNA methylation status were performed. We dichotomized methylation levels based on an optimized cut-off for patient classification. *CTLA4* DNA methylation significantly correlated with patients’ progression-free survival under ICB (*p* = 0.044, Fig. 3b). Patients with low methylation levels (below cut-off) showed significantly longer progression-free survival compared to patients with high methylation levels (above cut-off). A trend towards a better overall survival for patients with hypomethylated tumors could be observed, however not reaching statistical significance (*p* = 0.11, Fig. 3c).

### *CTLA4* promoter methylation correlates with occurrence of adverse events under ICB

To investigate whether *CTLA4* methylation was associated with immune-related adverse events (irAEs) under ICB, we gathered patient data on side effects according to the Common Terminology Criteria for Adverse Events (CTCAE). In our ICB-cohort, *N* = 1 patient developed a colitis (CTCAE Grade 2), *N* = 1 patient a hepatitis (CTCAE Grade 2), *N* = 2 patients a pneumonitis (CTCAE Grade 2), and *N* = 3 patients a dermatitis and/or mucositis (CTCAE Grade 1–2). In *N* = 2 patients, development of irAE remained unknown due to treatment continuation in external centers. No irAEs under ICB were observed in the remaining *N* = 21 patients. When dividing the patients into a group with irAEs and a group without irAEs, we detected significant higher mean levels of *CTLA4* methylation in the non-irAE group than in the irAE group (no irAEs: 61.99% [Standard Deviation (SD) 37.20], irAEs: 29.22% [SD 12.68], *p* = 0.002).

## Discussion

CTLA-4 is known to be expressed constitutively by regulatory T cells and to be upregulated upon activation by CD4^+^ and CD8^+^ T cells [12]. Yet, there is increasing evidence that CTLA-4 can also be expressed by tumor cells [25–27]. In this study, we show that CTLA-4 protein is also expressed by HNSCC cells. Both tumor infiltrating lymphocytes and HNSCCs displayed a heterogeneous CTLA-4 protein expression pattern. These findings are consistent with a previous study that immunohistochemically detected CTLA-4 protein expression in cancer cells of esophageal cancer [26]. Interestingly, HNSCC cells showed predominantly cytoplasmic CTLA-4 expression, but in rare cases, also nuclear expression. Studies on T cells could show that in the absence of its ligands, CTLA-4 is mainly found in intracellular compartments following clathrin-mediated endocytosis [28]. However, only few data are available concerning the regulation and function of CTLA-4 protein expression in tumor cells to date. In leukemic B cells, intracellular CTLA-4 expression is found and can be induced onto the cell surface upon co-culture with activated T cells [29]. Additionally, cytoplasmic staining of CTLA-4 protein has been described in diverse tumor entities [25–27], suggesting a similar mechanism of CTLA-4 endocytosis in tumor cells as observed in T cells. When comparing CTLA-4 expression in different HNSCC cases, we observed a potential gradient of expression in adjacent epithelium (increasing from basal to superficial epidermal layer) and in tumors (increasing from periphery to center of the tumor). In this study, we were not able to discriminate if the gradient is of potential biological interest, which is a limitation of the study. However, we suggest further investigation of the phenomenon in order to shed light onto a potential biological function.

Recently, emerging evidence of nuclear immune checkpoint expression and function has been reported, with focus on PD-L1. Nuclear PD-L1 has been shown to directly modulate and regulate gene transcription of several immune-related genes [30, 31]. Interestingly, PD-L1-regulated genes encounter on the one hand genes associated with inflammation resulting in the potential to cause an increased sensibility of the tumor to ICB, and on the other hand include other immune checkpoint genes, possibly leading to acquired ICB-resistance [31]. We recently described a nuclear expression of ICOS in melanoma, however, the biological significance remains unclear [32]. Similarly to the reports of PD-L1, other transmembrane receptors have been shown to be internalized and functioning as transcription factor, for example the epidermal growth factor receptor (EGFR) [33, 34]. Additionally, nuclear EGFR has been associated with therapy resistance to targeted therapy and histone deacetylase inhibitors in different cancers [34, 35]. In this study, we are to our knowledge the first to describe a nuclear expression of CTLA-4. If nuclear CTLA-4 harbors a biological function has yet to be elucidated, however, our finding encourage further investigation of nuclear CTLA-4 expression.

To date, the exact mode of action of anti-CTLA-4 antibodies and the role of tumor cell-intrinsic CTLA-4 expression with regard to response to ICB is only inadequately understood. In NSCLC cells, anti‐CTLA-4 antibody was able to induce PD‐L1 expression and to promote NSCLC cell proliferation and tumor growth in the absence of adaptive immunity, suggesting that tumor cell‐intrinsic CTLA-4 can regulate PD‐L1 expression and cell proliferation in cancer cells [36]. Mo et al. [37] could show, that treatment with interferon γ induces CTLA-4 protein expression in melanoma cells via activation of signal transducer and activator of transcription 1 (STAT1), which is recruited to the *CTLA4* promoter and modulates histone acetylation. Furthermore, the authors found CTLA-4 expression to be associated with the expression of the immune checkpoints PD-L1, hepatitis A virus cellular receptor 2 (HAVCR2, also known as T-cell immunoglobulin and mucin-domain containing-3 [TIM-3]), and lymphocyte activating 3 (LAG3) and to be correlated with response to anti-CTLA-4 immunotherapy.

In this study, we were not able to see correlations between *CTLA4* DNA methylation and protein expression in the heterogeneous tumor sample. However, at mRNA level we described significant correlations with *CTLA4* methylation in a prior study, but those were highly sequence-contextually dependent [24]. Since our analyses were conducted with heterogeneous tumor samples, absent correlations between *CTLA4* methylation and protein expression might not necessarily exclude a potential biological connection. Therefore, we analyzed the impact of the hypomethylating agent decitabine on CTLA-4 protein expression in five different human HNSCC cell lines. As expected, treatment with decitabine led to a decrease of *CTLA4* methylation and an increase of *CTLA4* mRNA and CTLA-4 protein expression, indicating an epigenetic regulation of *CTLA4* via DNA methylation in HNSCC. This finding is in line with various studies that described an epigenetic regulation of immune checkpoint genes via DNA methylation in diverse malignancies [19–23]. Interestingly, treatment with decitabine led to enlarged HNSCC cells with enlarged nucleus. Morphological changes including cell enlargement under treatment with decitabine have already been described in cancer cells [38] and have been interpreted as a sign of cellular senescence [39].

Although decitabine treatment led to decreased *CTLA4* methylation levels and increased mRNA and protein expression, we observed that *CTLA4* methylation in untreated cell lines did not correlate with the extent of CTLA-4 expression as even hypomethylated cell lines did not express CTLA-4 prior to decitabine treatment. This finding suggests that hypomethylation of the analyzed CpG sites is not sufficient to induce CTLA-4 expression in HNSCC cells. Since regulation of gene expression is complex, additional prerequisites seem necessary, e.g., demethylation of transactivating elements such as enhancers, and expression of transcription factors and cytokines. In melanoma, we have previously reported that *PD-L2* promoter methylation correlates with PD-L2 expression only after interferon γ stimulation, suggesting the necessity of promoter hypomethylation and cytokine stimulation to induce immune checkpoint expression [40]. However, as DNA methyltransferase inhibitors affect the whole genome, activate endogeneous retroviruses, and induce an interferon response [41], studying the significance of promoter methylation with regard to gene expression using decitabine is limited, particularly concerning immune genes.

To investigate the predictive value of *CTLA4* DNA methylation, we analyzed *CTLA4* DNA methylation levels in our cohort of patients with advanced or metastatic HNSCC prior to ICB. In our cohort, *CTLA4* promoter hypermethylation significantly correlated with poor therapy response. In addition, *CTLA4* promoter hypermethylation was significantly associated with shortened patients’ progression-free survival under anti-PD-1 therapy. These results are consistent with our previous findings showing that *CTLA4* promoter methylation predicts response to anti-CTLA-4 antibody therapy in melanoma [22], to anti-PD-1 antibody therapy in renal cell carcinoma [23], and predicts patients’ survival and response to anti–PD-1 or combined anti–PD-1 and anti–CTLA-4 ICB in melanoma [21]. In contrast, no significant correlation between *CTLA4* methylation and overall survival could be observed in the UKB Non-ICB cohort, which corresponds with our previous observations in the TCGA cohort [24], where *CTLA4* methylation was not significantly correlated with patients’ survival.

When investigating whether *CTLA4* methylation was associated with immune-related adverse events under ICB, we detected significant higher mean levels of *CTLA4* methylation in the non-irAE group than in the irAE group. So far, no correlation between the level of immune checkpoint expression, e.g. PD-1- and CTLA-4, and development of irAEs in patients receiving ICB has been established. However, occurrence of irAEs have been linked to improved cancer-related outcome in HNSCC [42]. Our results support these data, as patients with irAEs showed decreased mean levels of *CTLA4* methylation and *CTLA4* hypomethylation correlated with improved therapy response and longer progression-free survival under ICB in our patient cohort.

Consistent with our previous reports from HNSCC and melanoma [22, 24], we did not find significant correlations between *CTLA4* promoter methylation and CTLA-4 protein expression in our study. Potentially, inter- and intratumorally varying CTLA-4 turnover rates, posttranslational modifications (e.g. glycosylation), and the expression of diverse CTLA-4 isoforms hinder an accurate immunohistochemical detection of CTLA-4 protein [43]. In contrast, *CTLA4* DNA methylation can reliably be detected and quantified even in limited amounts of FFPE tissues, suggesting a higher biomarker performance of *CTLA4* methylation compared to CTLA-4 protein expression.

A limitation of our study is the small sample size of only *N* = 29 HNSCC in the UKB ICB cohort. However, since anti-PD-1 treatment only recently received regulatory approval for the treatment of HNSCC, the present cohort is of reasonable size. Due to the small sample size, we restricted our analysis to CpG sites that we have previously shown to harbour predictive values in melanoma and renal cell carcinoma [21–23]. Future investigations of larger ICB cohorts by means of powerful technologies, e.g., next generation bisulfite sequencing methods, are needed to analyzed all CpG sites within *CTLA4* and other relevant genes (e.g. *PD-1* and *PD-L1*) with regard to response prediction.

## Conclusions

In summary, *CTLA4* promoter methylation seems to play a role within the epigenetic regulation of CTLA-4 expression in HNSCC and its microenvironment and significantly correlates with therapy response and progression-free survival under anti-PD-1 therapy. As only few HNSCC patients respond to immune checkpoint treatments so far, mechanism-driven biomarkers that are able to predict therapy response are urgently needed. Our results warrant further testing of *CTLA4* promoter methylation as a predictive biomarker in clinical trials of HNSCC patients treated with anti-PD-1 and/or anti-CTLA-4 immunotherapy.

## Methods

### Patients

*UKB Non-ICB cohort*: A cohort of* N* = 138 histopathologically confirmed HNSCC patients without history of treatment with immune checkpoint blockade from an earlier study was included (Table 2) [44]. All patients were treated between 2011 and 2018 at the University Medical Center Bonn (UKB). The location of HNSCC was in *N* = 31 patients the larynx, *N* = 60 patients the oropharynx, *N* = 21 patients the hypopharynx,* N* = 18 patients the oral cavity, *N* = 4 the nasal cavity and paranasal sinuses, and *N* = 4 were carcinomas of unknown primary (CUP).

**Table 2:** Patients’ characteristics of the UKB Non-ICB cohort and the UKB ICB cohort

Characteristic	UKB Non-ICB cohort	UKB ICB cohort
	Number *N* (%)	Number* N* (%)
All patients	138	29
Age [years] (range)	62.5 (32–89)	62.3 (31–81)
Gender		
Female	24 (18.0)	9 (31.0)
Male	115 (82.7)	20 (69.0)
Stage (UICC)		
I	21 (15.2)	0 (0)
II	22 (15.9)	0 (0)
III	34 (24.6)	0 (0)
IV	61 (44.2)	29 (100)
Disease origin		
Oropharynx	60 (43.5)	10 (34.5)
Larynx	31 (22.5)	1 (3.4)
Hypopharynx	21 (15.2)	4 (13.8)
Oral cavity	18 (13.0)	8 (27.6)
Nasal cavity and paranasal sinuses	4 (2.9)	–
Carcinoma of unknown primary	4 (2.9)	6 (20.7)
HPV status in oropharyngeal carcinomas		
Positive	27 (45.0)	1 (10.0)
Negative	32 (53.3)	6 (60.0)
Unknown	1 (1.7)	3 (30.0)
Immune checkpoint inhibitor		
Cemiplimab	–	1 (3.4)
Nivolumab	–	27 (93.1)
Nivolumab + ipilimumab	–	1 (3.4)
Response to immune checkpoint blockade		
Partial response	–	4 (13.8)
Stable disease	–	7 (24.1)
Progressive disease	–	15 (51.7)
Unknown	–	3 (10.3)
Immune-related adverse events		
Colitis	–	1 (3.5)
Hepatitis	–	1 (3.5)
Pneumonitis	–	2 (6.9)
Dermatitis and/or mucositis	–	3 (10.3)
None	–	21 (72.4)
Unknown	–	2 (6.9)

Data include age, gender, stage according to UICC, site of the disease origin, human papillomavirus (HPV) status in oropharyngeal carcinomas, applied immune checkpoint inhibitor, response to ICB, and occurrence of immune-related adverse events

*UKB ICB cohort*: We further enrolled* N* = 29 histopathologically confirmed HNSCC of patients treated with either nivolumab (*N* = 27), cemiplimab (*N* = 1), or the combination of nivolumab and ipilimumab (*N* = 1) at the UKB between 2015 and 2020. Survival data were available from all *N* = 29 patients. Survival analyses were performed regarding progression-free survival and overall survival. Therapy response data determined by response evaluation criteria in solid tumors (RECIST 1.1) were available for 26 patients. Immune-related adverse events (irAEs) were recorded according to the Common Terminology Criteria for Adverse Events (CTCAE, version 5.0). Patient inclusion and sample analyses at the University Hospital Bonn were approved by the Institutional Review Board (IRB) of the University Hospital Bonn (vote 187/16). Informed consent was obtained from all individual participants included in the study. Patients’ characteristics are shown in Table 2.

*Tonsillar tissue:* Fresh frozen tonsillar tissues were received from the BioBank Bonn of the UKB.

### *CTLA4* methylation analysis

The tumor was identified by a pathologist based on a hematoxylin and eosin (H&E) slide and the respective area was scraped from adjacent 10 µm sections using a scalpel. The tissue was lysed and bisulfite-converted as previously described [45]. DNA concentration was quantified by UV spectroscopy using a N60 NanoPhotometer (Implen GmbH, Munich, Germany). We used a quantitative methylation-specific PCR (qMSP) for the quantification of the total amount of methylated *CTLA4* copies, as previously analyzed in different tumor entities [21–23]. A reference assay that amplifies a CpG-free region within the *ACTB* gene locus was duplexed with the *CTLA4* qMSP assay in order to quantify the total DNA amount. Quantitative Methylation Scores (QMS) were calculated using a modified ΔΔCT method as previously described [46]. Bisulfite-converted CpGenome Universal Methylated DNA (Merck KGaA, Darmstadt, Germany, cat. no. S7821) was used as calibrator sample. We applied 30 ng template DNA per PCR reaction, and each sample was measured in triplicate. The PCR buffer composition and cycling conditions are reported elsewhere [21, 45]. The oligonucleotides we synthesized by biomers.net (Ulm, Germany; *CTLA4* qMSP: 6-FAM-aagtcgtgggtttagttgttac-BHQ-1, probe; gtttttttgttttggttttacga, reverse primer; tacttaaaattatcttttcgacg, forward primer; *ACTB* reference assay: Atto-647N-accaccacccaacacacaataacaaacaca-BHQ-2, probe; ggaggaggtttagtaagttttttg, reverse primer; cccttaaaaattacaaaaaccacaa, forward primer).

### mRNA expression analysis

qRT-PCR was used to determine *CTLA4* mRNA levels in HNSCC cell lines and tonsillar tissues. Quantification of total mRNA was achieved using five housekeeping genes (*ACTB*, *ALAS1*, *GAPDH*, *HPRT1*, *SDHA*). We performed RNA extraction using the NucleoSpin RNA Mini kit (Macherey Nagel, Düren, Germany, cat. no. 740955) according to the manufacturer’s instructions. We used the HiScript II Q RT SuperMix for qPCR (Vazyme, Nanjing, China, cat. no. R222) for cDNA synthesis. Quantification by qRT-PCR was carried out using PCR buffer conditions as described above. Oligonucleotide specifications and PCR annealing temperatures are listed below (Table 3). PCR was performed at 20 min / 95 °C and 45 cycles of [assay-specific annealing temperature / 60 s, 95 °C / 15 s]. ΔCT values were calculated (ΔCT_sample_ = CT_reference genes_ – CT_*CTLA4*_) using mean CT values of all five reference genes (CT_reference genes_).

**Table 3:** Oligonucleotide sequences, modifications, final concentrations, and assay-specific annealing temperatures

Gene	Forward Primer (5′ → 3 ′)	Reverse Primer (5′ → 3 ′)	Probe (5′ → 3 ′)^¥^	Annealing temperature
*CTLA4*	0.4 µM CTCATGTACCCACCGCCATACT	0.4 µM TTGATGGGAATAAAATAAGGCTGAA	0.2 µM 6-FAM-CAGATTTATGTAATTGATCCAGAACCGTGCC-BHQ-1	62 °C
*ACTB*	0.2 µM ATGTGGCCGAGGACTTTGATT	0.2 µM AGTGGGGTGGCTTTTAGGATG	0.16 µM HEX-GAAATRMGTKGTTACAGGAAGTCCCT-BHQ-1	58 °C
*ALAS1*	0.2 µM TAATGACTACCTAGGAATGAGTCG	0.2 µM CCATGTTGTTTCAAAGTGTCCA	0.16 µM 6-FAM-TAACTGCCCCACACACCCGT-BHQ-1	62 °C
*GAPDH*	0.2 µM TGCACCACCAACTGCTTAGC	0.2 µM GGCATGGACTGTGGTCATGAG	0.16 µM 6-FAM-CTGGCCAAGGTCATCCATGACAACT-BHQ-1	58 °C
*SDHA*	0.2 µM TCGCTCTTGGACCTGGT	0.2 µM TGGAGGGACTTTATCTCCAG	0.16 µM 6-FAM-ATCGAAGAGTCATGCAGGCC-BHQ-1	62 °C
*HPRT1*	0.2 µM TGACACTGGCAAAACAATGCA	0.2 µM GGTCCTTTTCACCAGCAAGCT	0.16 µM 6-FAM-TGCTTTCCTTGGTCAGGCAGTAT-BHQ-1	62 °C

^¥^wobble bases: R–A/G, M–A/C, K–G/T

### Immunohistochemistry

CTLA-4 protein expression was assessed on whole slides via immunohistochemistry (IHC) in a subset (*N* = 113) of the UKB Non-ICB cohort as described earlier [22]. In brief, FFPE tumor tissues sections of 4 µm thickness were deparaffinizated and incubated with Target Retrieval Solution (pH6, Dako/ Agilent Technologies, Inc., Santa Clara, CA, USA) at 100 °C for 10 min. Sections were subsequently washed with TBS. Primary CTLA-4 antibody (dilution 1:50, mouse monoclonal antibody, clone BSB-88, cat# BSB 2884, RRID:AB_2762365, Bio SB, Santa Barbara, CA, USA) was added, incubated at 4 °C overnight, and washed with 550 mM TBS. REAL Detection System Alkaline Phosphatase/RED (Dako/Agilent Technologies) was utilized to visualize bounded primary antibody according to the manufacturer’s protocol. Tonsillar tissue was used as a positive control. Histoscore (H-score) was applied to quantify CTLA-4 expression in tumor cells and was calculated according to Detre et al*.* [47] as followed: 10 fields were chosen at random at 400 × magnification, and the staining intensity in the malignant cells was scored as 0, 1, 2, or 3 for the presence of negative, weak, intermediate, and strong pink staining. We counted the number of cells in each field and the number of cells stained at each intensity and calculated an average percentage of positive staining with the following formula: H-score = (% of cells stained at intensity category 1 × 1) + (% of cells stained at intensity category 2 × 2) + (% of cells stained at intensity category 3 × 3). A final H-score between 0 and 300 was obtained for each staining, and the average of H-score for all the cases calculated. Cases with H-score higher than average were regarded as high expression and those with H-score equal or less than average as low expression. CD3^+^, CD4^+^, CD8^+^, and CD45^+^ immune cell infiltrates were immunohistochemically quantified in the course of our previous study [44].

### Tumor cell lines and 5‐aza‐2‐deoxycytidine treatment

We included data from *N* = 2 HPV-positive (FaDu/HTB-43 [RRID:CVCL_1218], UPCI-SCC-152/CRL-3240 [RRID: CVCL_C058]) and *N* = 3 HPV-negative HNSCC cell lines (HSC-3/ SCC-193 [RRID: CVCL_1288], SCC-25/CRL-1628 [RRID: CVCL_1682], Detroit 562/ CLL-138 [RRID: CVCL_ 1171]). HSC-3 was obtained from Merck (Darmstadt, Germany). All other cell lines were purchased from ATCC (Manassas, Virginia, USA). Mycoplasma contamination testing was performed regularly. The cell lines were grown adherent and maintained in complete DMEM medium (cat. no. 12430054, Thermo Fisher Scientific, Waltham, MA, USA) supplemented with 10% [v/v] fetal bovine serum (FBS, heat inactivated, cat. no. FBS. S 0615HI, Bio&SELL GmbH, Nuremburg, Germany), 1X MEM (Minimum Essential Medium) Non-Essential Amino Acids Solution (100X stock, cat. no. 11140035, Thermo Fisher Scientific), 1 mM 2-mercaptoethanol (cat. no. 21985023, Thermo Fisher Scientific), 100 U/ml penicillin and streptomycin (10,000 U/ml stock, cat. no. 15140122, Thermo Fisher Scientific), and 1 mM sodium pyruvate (100 mM stock, cat. no. 11360070, Thermo Fisher Scientific). The cells were either left untreated for seven days or treated with demethylating 5‐aza‐2‐deoxycytidine (decitabine, 5‐Aza-dC). For decitabine treatment, 10 μM 5‐aza‐dC (cat. no. ab120842, Abcam, Cambridge, UK) were supplemented to the growth medium every 24 h over a seven days period.

### Flow cytometry

HNSCC cell line pellets were washed with flow cytometry buffer (1X Dulbecco’s Phosphate Buffered Saline [cat. no. 14190094, Thermo Fisher Scientific], 4% [v/v] FBS, 2 mM ethylenediaminetetraacetic acid [EDTA]). Single cell suspensions were stained with the following fluorochrome-conjugated antibodies: LIVE/DEAD™ Fixable Near-IR Dead Cell Stain Kit (cat. no. L10119, Thermo Fisher Scientific, 1:1,000 in flow cytometry buffer) and CTLA-4 mouse monoclonal antibody (dilution 1:100 in flow cytometry buffer, mouse monoclonal antibody, clone BSB-88, labeled with secondary goat anti-mouse IgG antibody [cat. no. A-11001, Thermo Fisher Scientific]). The liquid phase was removed and the cell pellet resuspended in 300 µl flow cytometry buffer. Flow cytometry data were acquired with a FACSCanto™ Flow Cytometer (Becton, Dickinson and Company, NJ, USA) and analyzed with FlowJo software (version 10.8.0, Becton, Dickinson and Company).

### Statistics

Statistical analyses were conducted using IBM SPSS Statistics version 27 (IBM, Armonk, NY, USA). Correlations were calculated using Spearman’s rank correlation (Spearman’s *ρ*). Group comparisons were made using *t-*tests or Kruskal–Wallis (> 2 groups) tests. Survival analyses were performed using Kaplan–Meier models and Cox proportional hazards analysis. Progression-free survival was defined as the time between the first application of ICB and the date of documented disease progression. Overall survival was defined as the time between initial diagnosis (UKB Non-ICB cohort) or initiation of ICB (UKB ICB cohort) and death or last contact, respectively. For Kaplan–Meier analyses, methylation levels were dichotomized based on an optimized cutoff. Cut-off optimization was performed with regard to *p*-values. *P*-values refer to log-rank test. Two-sided *p*-values < 0.05 were considered statistically significant.

## Data Availability

The datasets generated during and/or analyzed during the current study are available from the corresponding author on reasonable request.
